# Pharmacological and molecular analysis of the effects of Huangqi Jianzhong decoction on proliferation and apoptosis in GES-1 cells infected with *H. pylori*

**DOI:** 10.3389/fphar.2022.1009705

**Published:** 2022-09-29

**Authors:** Jingnan Hu, Tao He, Jianfang Liu, Sujie Jia, Bolin Li, Weichao Xu, Man Liao, Lifang Guo

**Affiliations:** ^1^ Hebei Province Hospital of Chinese Medicine, Shijiazhuang, China; ^2^ Hebei Industrial Technology Institute for Traditional Chinese Medicine Preparation, Shijiazhuang, China

**Keywords:** Huangqi Jianzhong decoction, *Helicobacter pylori*, human normal gastric mucosa epithelial cells, cell proliferation, apoptosis, network pharmacology

## Abstract

**Background:** Infection with *Helicobacter pylori* (*H. pylori*) can cause chronic gastritis and other digestive tract diseases, and represents a public health concern. Current anti-*H. pylori* treatment can result in antibiotic resistance and other adverse reactions. Huangqi Jianzhong decoction (HQJZD) is a prescription form of traditional Chinese medicine for chronic gastritis that increases probiotics and inhibits *H. pylori*. In this study, its anti-bacterial activity against *H. pylori* receives a preliminary evaluation, and a pharmacology analysis is performed to predict its underlying mechanisms.

**Methods:** Human GES-1 cells are divided into a blank control group, a model group, a HQJZD low-dose (2.08 mg·mL^−1^), a high-dose group (4.16 mg·mL^−1^), and a positive control group (amoxicillin, 5 μg·mL^−1^). After culture, the CCK-8 method is used to detect cell viability; flow cytometry is used to detect cell apoptosis rate; and RT-qPCR is used to detect the expression of mRNA virulence factors, including HpPrtC, OPiA, IceA1, and BabA2. Network pharmacology analysis and molecular docking were performed to explore the mechanisms of HQJZD in treating *H. pylori* gastritis, based on its anti-*H. pylori* infection effect.

**Results:** We noted lower cell survival rates in the model group, but higher apoptosis rates and mRNA expressions of HpPrtC, OPiA, IceA1, and BabA2 than in the control group (*p* < 0.05). Compared to the model group, the cell survival rate of each dosage group of Huangqi Jianzhong decoction and the positive control group increased significantly, while the apoptosis rate and the mRNA expressions of HpPrtC, OPiA, IceA1, and BabA2 were decreased significantly. The effect in each HQJZD group was dose-dependent (*p* < 0.05). Network pharmacological analysis involving 159 signaling pathways was used to screen 6 key active components of HQJZD and 102 potential target proteins for the treatment of *H. pylori*-related gastritis. The molecular docking results revealed that the 6 active compounds had a strong binding ability with the target proteins of ALB, IL-6, AKT1, IL-1B, and JUN.

**Conclusion:** HQJZD effectively increases the proliferation rate of human GES-1 cells after infection, while reducing the level of apoptosis. The mechanism may be related to multiple components, multiple targets and pathways, which provides a scientific basis for further elucidating the mechanism of action, the pharmacodynamic material basis, and the clinical application of HQJZD against *H. pylori* infection.

## Introduction

*Helicobacter pylori* (*H. pylori*) is a helix-shaped Gram-negative bacteria that can easily adapt to the gastric environment of the stomach and is found in almost half of the population worldwide (Ren et al., 2022). It damages the gastric epithelial cells by adhering to the gastric mucosa and injecting virulence factors into the epithelial cells, triggering a chronic inflammatory response that further expands gastric tissue damage (Ku et al., 2022). Several reports suggest that *H. pylori* infection can cause chronic gastritis, intestinal metaplasia, gastric adenocarcinoma, and dyspepsia (Graham et al., 2021; Miller and Williams, 2021). Current conventional treatments for *H. pylori* mainly include triple or quadruple therapy (a proton pump inhibitor and two antibiotics, and a bismuth agent), which directly sterilize and protect the gastric mucosa, and neutralize the gastric acids. Such treatment can easily cause antibiotic resistance or other adverse reactions (Zhang et al., 2019). Traditional herbs in Chinese medicine have gained recent attention in the clinical eradication of *H. pylori*, since finding an effective treatment modality to reduce the inflammatory responses of the body is of such great significance.

Huangqi Jianzhong decoction (HQJZD) is a traditional Chinese herbal formula that consists of seven different herbs: *Radix Astragali* (Huangqi), *Paeoniae Radix Alba* (Baishao), *Ramulus Cinnamomi* (Guizhi), *Rhizoma Zingiberis Recens* (Shengjiang), *Radix Glycyrrhizae* (Gancao), *Fructus Jujube* (Dazao), and *Saccharum Granorum* (Yitang) (Mai et al., 2022). The use of HQJZD was first recorded in the *Synopsis of Prescriptions of the Golden Chamber* by Zhongjing Zhang, and is mainly used in clinical practice for treating gastrointestinal diseases, such as chronic gastritis, peptic ulcers, and atrophic gastritis, and has also proven efficacious against *H. pylori*-related gastritis (Zhang et al., 2022). In addition, studies have shown that *Astragalus membranaceus* in Huangqi Jianzhong decoction can enhance the immunity of the body and inhibit the propagation of harmful bacteria by regulating the intestinal flora and increasing the species and number of probiotics (Chen et al., 2022). However, the underlying molecular mechanism of action against *H. pylori* infection is unclear.

Network pharmacology, based on integrity of component and targets, is an effective method for studying the molecular mechanisms of traditional Chinese medicine. The interaction between the disease and complex components in traditional Chinese medicine is explained from the perspective of a biological network, which is in line with the characteristics of “multiple targets” and “multiple pathways” of traditional Chinese medicine. The Gene Expression Omnibus (GEO) is a public database (supported by the National Center for Biotechnology Information), that stores high-throughput gene expression microarrays and datasets (Clough and Barrett, 2016), used to explore the molecular pathways of diseases at the gene level. In this study, we combined gene chips from the GEO database with network pharmacology and molecular docking to explore the molecular mechanism of HQJZD in treating *H. pylori*-related gastritis. We assume the results will provide a referential basis for further experimental research aimed at developing the classical prescriptions and will thereby help in clinical application.

**FIGURE 1:**
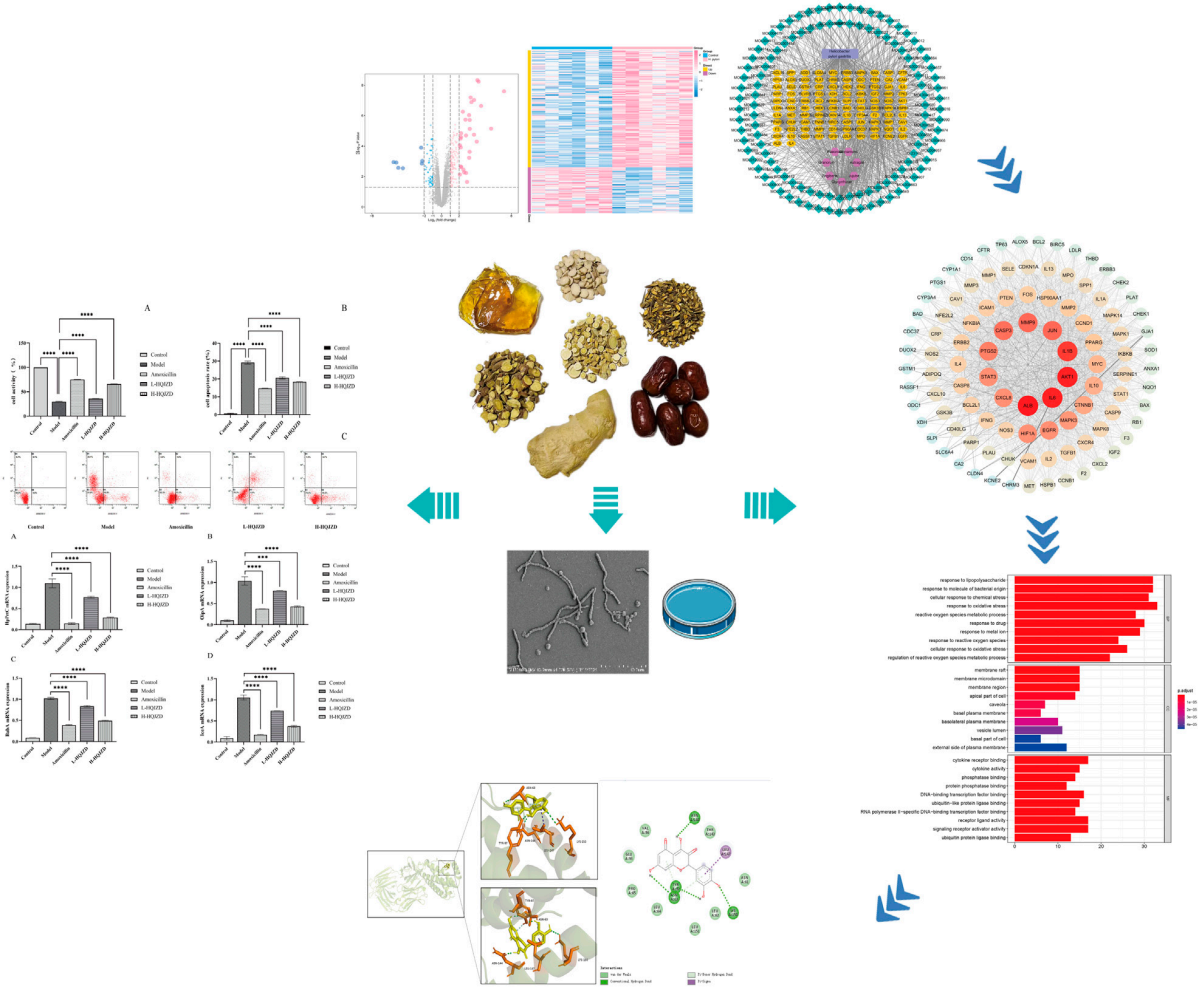
Overall design of the experiment.

In this study, we established a model of GES-1 cells based on network pharmacology and molecular docking, in order to evaluate the anti-*H. pylori* effects of HQJZD and explore its mechanisms of action. The aims are to accelerate future research and promote the clinical application of HQJZD as a complementary therapy against *H. pylori* infection. The study design of our experiment is shown in Figure 1.

## Materials and methods

### Chemicals and reagents

We used RPMI1640 medium (HyClone, American), trypsin, a cell counting kit-8 (CCK-8), an annexin V-FITC/PI apoptosis assay kit, and 0.1% diethylpyrocarbonate (DEPC)-water (all purchased from Beijing Solarbio Science and Technology Co., Ltd.). We also used fetal calf serum (Sijiqing Co. Ltd. Lot 11011-8611), TRIzol (Ambion [American]), chloroform, isopropanol, and absolute ethyl alcohol (all from Tianjin Kemiou Chemical Reagent Co. Ltd.). The RNA extraction kit (lot TCF-514123) was obtained from Proteintech Group Inc. (Wuhan, China). The HiFiScript gDNARemoval cDNA Synthesis Kit (lot CW2582M) was obtained from Jiangsu Cowin Biotech Co., Ltd. ChemoHS qPCR Mix (None ROX) (lot MQ00101S) was obtained from Monad Biotech Co., Ltd. (Wuhan, China).

### Sample preparation

Medicinal herbs of HQJZD were obtained from the pharmacy department of Hebei Province Hospital of Chinese Medicine. We soaked 500 g of medicinal herbs in 1000 ml of distilled water for 60 min, which was then condensed and refluxed for 120 min. The resulting filtrate was concentrated under reduced pressure in a rotary evaporator at 55°C to obtain 200 ml HQJZD of 8.32 mg·mL^−1^ concentration. Finally, this solution was diluted in water to reach concentrations of 4.16 mg·mL^−1^ and 2.08 mg·mL^−1^.

### Cell culture and grouping

The international standard strain of *H. pylori*, ATCC43504 (obtained from Beijing Biobw Biotechnology Company; NCTC 11637), and the human normal gastric epithelial cell line (GES‐1) (Wuhan Pu-nuo-sai Life Technology Co. Ltd.) were stored at −80°C. The frozen GES-1 cells were taken from the liquid nitrogen tank and immediately placed into a 37°C water bath for cell resuscitation. Resuscitated cells were placed in Roswell Park Memorial Institute (RPMI-1640) medium and split using cell culture dishes. They were then placed in an incubator for culturing, which was supplemented with 10% fetal bovine serum at 37°C with 5% CO_2_. The cell coverage rate was observed after culturing the cells for 24 h. When the cell coverage reached 70%, the medium was replaced and trypsin was added.

According to the intervention doses, and whether there was *H. pylori* infection (multiplicity of infection [MOI] at 200:1), cells were divided into a blank control group (normal human GES-1); a model group (HP infection + normal human GES-1); an HQJZD low-dose group (HP infection + normal human GES-1 + HQJZD 2.08 mg·mL^−1^); a HQJZD high-dose group (HP infection + normal human GES-1 + HQJZD 4.16 mg·mL^−1^); and a positive control group (HP infection + normal human GES-1 + amoxicillin 5 μg·mL^−1^).

### Evaluation of anti-*H. pylori* activity

#### Detection of cell activity

For detection of cell activity, 1 ml of trypsin was added when the cells were in the log growth phase. Cells were completely suspended after being slightly blown for 2 min. Suspended cells were transferred to 1.5 ml centrifuge tubes and were centrifuged at 2000 rpm for 10 min. The supernatant was discarded and 1 ml RPMI1640 medium was added to fully suspend the cells. Then 100 μL of this cell suspension was added to a 96-well plate, followed by incubation in a new RPMI-1640 medium for 24 h. The culture medium was then discarded and 100 μL of culture medium and 10 μL of CCK-8 solution were added and incubated continuously for 3 h. The procedure was duplicated in six wells for each group. The cell survival was calculated by analyzing their absorbance at 450 nm (Bio-Rad microplate reader), and this was repeated in sets of three.

#### Detection of cell apoptosis

The protective effect of HQJZD was observed on GES-1 cells infected with *H. pylori* by detecting the cell apoptosis rate. 3 ml of binding buffer (10×) was diluted to 30 ml with 27 ml deionized water. The cells were then re-suspended in 1 ml binding buffer (1×) and centrifuged for 10 min at the rate of 300 × *g*. The supernatant was discarded and the cells were re-suspended with 1 ml binding buffer (1×) at a density adjusted to 1×10^6^ cells/mL. Then, 100 μL of cell suspension and 5 μL of annexin V-FITC were added to each tube and gently mixed and incubated for 10 min at room temperature in the dark. Then, 5 μL of propidium iodide (PI) was added, followed by incubation for 5 min at room temperature in the dark. Finally, 390 μL PBS (1×) was added and gently mixed, and the cell apoptosis rate was detected by flow cytometry.

#### Expression of virulence factors

We used a quantitative polymerase chain reaction (qPCR) to detect the relative virulence factors of *H. pylori*. The primers for amplification were designed to amplify two different fragments located at the genes: HpPrtC, OPiA, IceA1, and BabA2.

The culture solution was discarded in a sterile Petri dish. The total amount of RNA extracted from the cells was inverted into cDNA and stored at −80°C for future use. The configuration system was set as follows: 2.25 μL of primer, 3.0 μL of cDNA, 17.25 μL of SYBR Green qPCR SuperMix, and 7.5 μL of enzymatic water. Six replicate wells were set up for each sample. The conditions for carrying out qPCR consisted of an initial melting cycle for 12 min at 94°C, followed by 35 cycles of amplification at 96°C for 12 s, 62°C for 25s, and 72°C for 40 sec. Dissociation curves were utilized to estimate the specific melting temperature of both the amplicon and the 2^–ΔΔ Ct^ method, which helped analyze the results of real-time qPCR. The primer sequences are listed as follows:

**Table:** 

Gene	Primer sequence	Length of PCR products
HpPrtC	F: 5′-GTAAGGCCGAAATCAAGCA-3′	204 bp
	R: 5′-TATCCACGCAAGCGAATGT-3′	
OPiA	F: 5′-GTGGCGTTGGTTCTGTTC-3′	307 bp
	R: 5′-GTGCGACTCTTGACTTGATT-3′	
IceA1	F: 5′-GTGTTTTTAACCAAAGTATC-3′	247 bp
	R: 5′-CTATAGCCAGTCTCTTTGCA-3′	
BabA2	F: 5′-CTTCTGACGTGTGGACTTAT-3′	242 bp
	R: 5′-CAT​CCT​CAC​TAA​CAT​GTT​GA-3′	

### Network pharmacological analysis

#### Acquisition of active ingredients and targets

The chemical constituents and targets of *Radix Astragali*, *Paeoniae Radix Alba*, *Ramulus cinnamomi*, *Rhizoma Zingiberis Recens*, *Radix glycyrrhizae*, and *Fructus Jujube* were identified using the TCMSP database (https://tcmspw.com/tcmsp.php) (Ru et al., 2014), and the screening conditions for active components was set as OB ≥ 30% and DL ≥ 0.18. Since *S. granorum* was not included in the TCMSP database, its composition was determined using the Batman-TCM database (http://bionet.ncpsb.org.cn/batman-tcm/) (Liu et al., 2016), and these results were supplemented to obtain the targets of action as the component conditional on a score of >40. All targets were put into the UniProt database (https://www.uniprot.org/) (UniProt Consortium, 2019) to remove non-human-derived species, as well as non-validated targets, and were summarized to obtain the active ingredient targets of HQJZD.

#### Acquisition of disease targets

The series matrix gene chip data file of GSE27411 was downloaded from the NCBI GEO public database, and differential genes from healthy individuals and patients with *H. pylori-*induced gastritis were selected, using the R language conditional on | logFC >1 | and *p* < 0.05. Meanwhile, “gastritis” and “*Helicobacter pylori* infection” were set as the key words for searching in GeneCards (Rebhan et al., 1997), OMIM, DisGenet, and other databases. The differential genes were selected by the GEO gene chips and integrated with the disease genes obtained from the other databases. After data cleaning and de-duplication, the related disease targets were obtained. The selected targets of components and diseases were uploaded into the Venny 2.1 software for mapping, and the intersection was used to obtain common targets of HQJZD for treating *H. pylori* gastritis.

#### Construction of an “active ingredients–disease–targets” network

Information regarding common targets of active ingredients of HQJZD in treating *H. pylori* gastritis was imported into Cytoscape 3.7.2 (Doncheva et al., 2019) software to construct the “active ingredients–disease–targets” network, which was topologically analyzed using the “Network Analyzer” tool. The degrees of the ingredients were ranked according to the value of the degree. Higher degrees were synonymous with importance, and under the condition that their degree value was greater than the average, active ingredients were selected as the key ingredients for subsequent studies.

### Protein–protein interaction

The common targets were entered into the String database (https://string-db.org/cgi/input.pl) (Szklarczyk et al., 2019). The minimum required interaction score was set as “0.4”; the biological species was set as “*Homo sapiens*”; and the protein–protein interaction (PPI) network was then constructed to obtain the PPI network. This was imported into Cystoscape 3.7.2 to obtain the core targets for HQJZD in treating *H. pylori* gastritis by topological analysis, and the degree values were ranked accordingly.

#### Gene enrichment analysis and “component–disease–target–pathway” network construction

Common targets of drugs and diseases were entered into the David database (Huang et al., 2009), and a biological process (BP), molecular function (MF), cell component (CC) enrichment, and KEGG pathway enrichment of GO were performed, conditional on a *p* value < 0.05. To further reflect the characteristics of the multi-component, multi-target action of traditional Chinese medicine in the treatment of diseases, Cytoscape 3.7.2 software was used to draw a “component–disease–target–pathway” network diagram.

### Molecular docking validation

Six key components and the top five core targets of HQJZD were used as ligands and receptors, respectively, for molecular docking. The sdf three-dimensional structure of key component compounds was downloaded from the PubChem database (https://pubchem.ncbi.nlm.nih.gov/) (Kim et al., 2021) and saved, and the core target protein structure was downloaded from the PDB database (https://www.rcsb.org/) (Goodsell et al., 2020). The proteins, ligands, and small molecules were dehydrated and hydrogenized to optimize them using the AutoDock Tools software, and they were docked using the AutoDock Vina software to predict the binding performance of proteins and ligands and hence to verify our accuracy. The docking results were visualized and displayed using Pymol software.

### Statistical analysis

All statistical analyses were performed using the SPSS software version 23.0 and the measurement data were presented as ‾x ± s. The differences between multiple groups were performed by single-factor analysis of variance (one-way ANOVA test, *p* < 0.05).

## Results

### Effects of HQJZD on GES-1 cells with *H. pylori* infection

**FIGURE 2:**
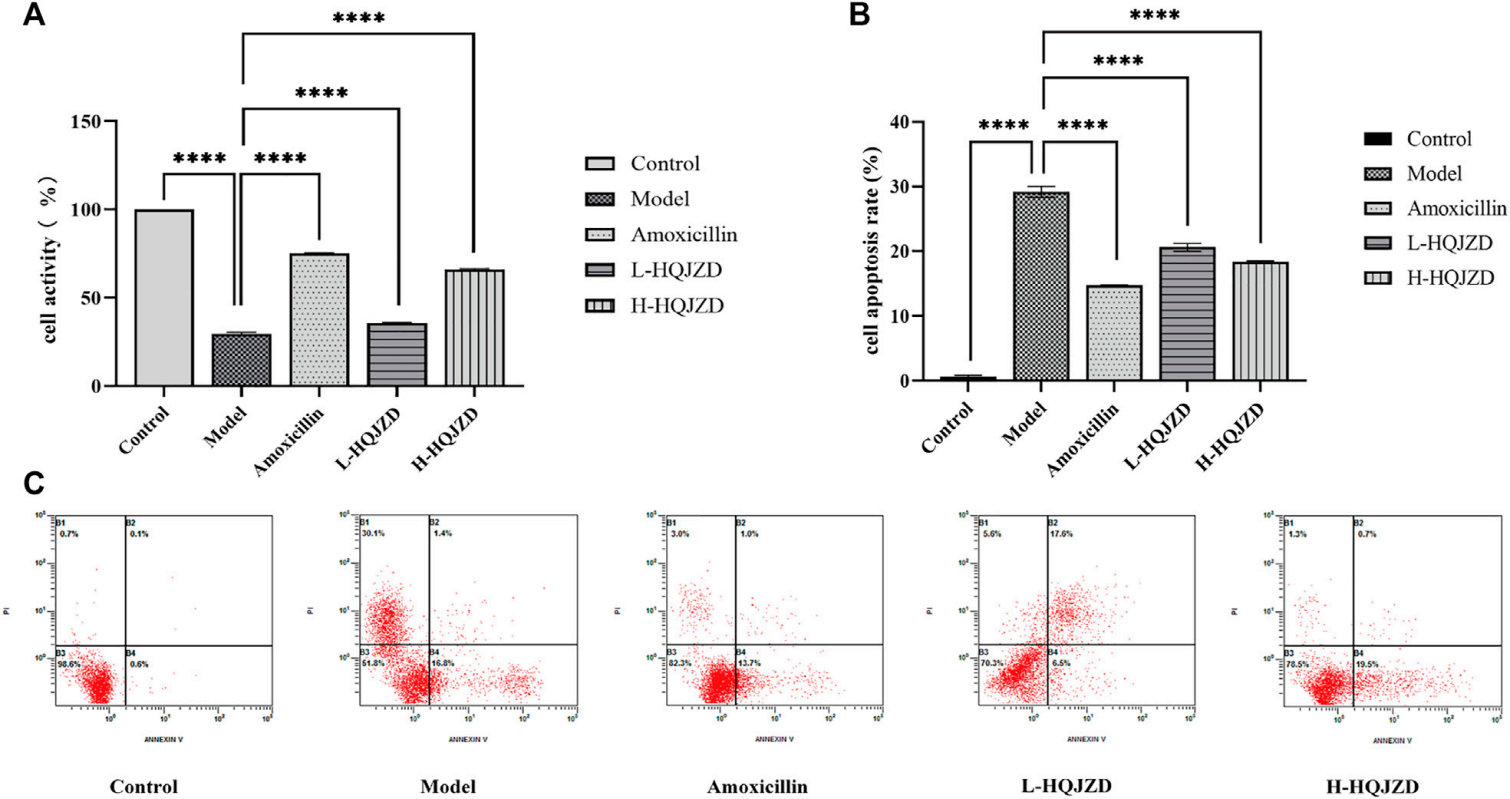
Effect of HQJZD on proliferation and apoptosis of GES-1 cells infected with *H. pylori*. **(A)** Cell activity of GES-1 cells; **(B)** cell apoptosis rate of GES-1 cells; and **(C)** graph of the apoptosis assay of the different groups.

The cell survival rate of the model group was significantly reduced compared to the blank control group, thereby indicating the proliferation of GES-1 cells that had been inhibited with *H. pylori* infection (*p* < 0.0001). Compared to the *H. pylori* infection group, adding both medium- and high-quality HQJZD improved cell viability and significantly reduced inhibition of *H. pylori* in the proliferation of GES-1 cells (*p* < 0.0001). Compared to the amoxicillin intervention group, the protective effects on GES-1 cell proliferation in the high-dose HQJZD group were closer, although the cell viability of HQJZD intervention groups was lower than in the positive drug group (Figure 2A).

For the early apoptosis (lower right quadrant) and the late apoptosis (upper right quadrant) of each group, cell apoptosis was calculated to evaluate the protective effects of HQJZD on GES-1 cells that were infected by *H. pylori* (Figure 2C). Compared to the blank control group, apoptosis rates in the model group were significantly higher (*p* < 0.0001), and *H. pylori* infection significantly induced apoptosis in GES-1 cells. After the intervention of HQJZD, both low and high concentrations of HQJZD significantly reduced apoptosis in GES-1 cells induced by *H. pylori* (*p* < 0.0001), compared to the model group (Figure 2B).

**FIGURE 3:**
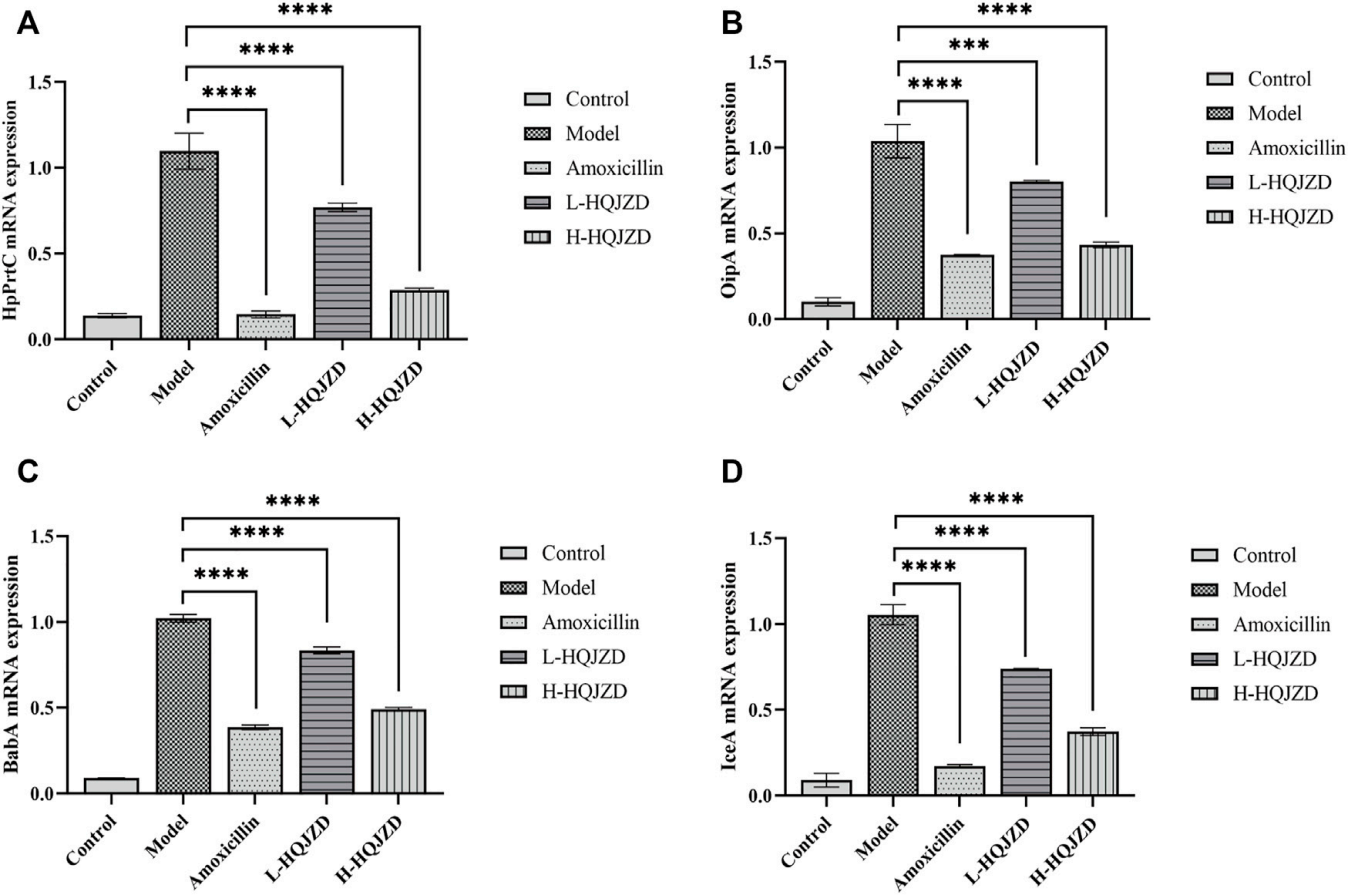
Effect of HQJZD on the expression of HpPrtC, OPiA, IceA1, and BabA2 mRNA in GES-1 cells infected with *H. pylori*. **(A)** mRNA expression of HpPrtC; **(B)** mRNA expression of OPiA; **(C)** mRNA expression of BabA2; and **(D)** mRNA expression of IceA1.

We used qPCR to assess the effects of HQJZD on the expression of virulence factors of *H. pylori*, and calculated the relative expressions of HpPrtC, OPiA, IceA1, and BabA2 mRNA. Compared to the blank group, *H. pylori* infection could significantly increase the expression of HpPrtC, OPiA, IceA1, and BabA2 mRNA in GES-1 cells (*p* < 0.001). As seen in Figure 3, compared to the *H. pylori* infection group, adding HQJZD at lower and higher concentrations could significantly reduce the expressions of HpPrtC, OPiA, IceA1, and BabA2 mRNA in GES-1 cells (*p* < 0.001). There was no significant difference in the mRNA expression for OPiA between the positive control group and the HQJZD high-dose group (*p >* 0.05).

### Mechanism prediction of HQJZD in treating *H. pylori* gastritis

#### Active ingredients and potential targets of HQJZD in the treatment of *H. pylori* gastritis

A total of 171 active components of HQJZD were obtained from the database search and screening under the conditions of OB ≥ 30% and DL ≥ 0.18, including 13 components from *Paeoniae Radix Alba*, 29 from *Fructus Jujube*, 93 from *Radix Glycyrrhizae*, 7 from *Ramulus Cinnamomi*, 20 from *Radix Astragali*, 5 from *Rhizoma Zingiberis Recens*, and 4 from *Saccharum Granorum*. After deleting the repeats in the active ingredients, and the active ingredients for which no corresponding target could be found in the TCMSP database, 128 active ingredients were finally obtained, of which 11 were common active ingredients (Table 1). After de-reprocessing, using the “related targets” module in the TCMSP database, 318 corresponding targets were obtained for the active ingredients of HQJZD.

**TABLE 1:** Information on common active ingredients of Huangqi Jianzhong decoction.

MOL ID	Common active ingredient	Attribution of medicinal materials	Number of targets	OB (%)	DL
MOL000211	Mairin	*Radix Astragali*, *Paeoniae Radix Alba*, *Radix Glycyrrhizae*, *Fructus Jujube*	1	55.38	0.78
MOL000358	β-Sitosterol	*Paeoniae Radix Alba*, *Ramulus Cinnamomi*, *Rhizoma Zingiberis Recens*, *Fructus Jujube*	38	36.91	0.75
MOL000359	Sitosterol	*Paeoniae Radix Alba*, *Radix Glycyrrhizae*, *Ramulus Cinnamomi*	3	36.91	0.75
MOL000422	Kaempferol	*Radix Astragali*, *Paeoniae Radix Alba, Radix Glycyrrhizae*	61	41.88	0.24
MOL000492	(+)-Catechin	*Paeoniae Radix Alba*, *Ramulus Cinnamomi*, *Fructus Jujube*	9	54.83	0.24
MOL000239	Jaranol	*Radix Astragali*, *Radix Glycyrrhizae*	14	50.83	0.29
MOL000354	Isorhamnetin	*Radix Astragali*, *Radix Glycyrrhizae*	37	49.60	0.31
MOL000392	Formononetin	*Radix Astragali*, *Radix Glycyrrhizae*	40	69.67	0.21
MOL000417	Calycosin	*Radix Astragali*, *Radix Glycyrrhizae*	24	47.75	0.24
MOL000098	Quercetin	*Radix Astragali*, *Radix Glycyrrhizae*, *Fructus Jujube*	142	46.43	0.28
MOL000449	Stigmasterol	*Rhizoma Zingiberis Recens*, *Fructus Jujube*	30	43.83	0.76

**FIGURE 4:**
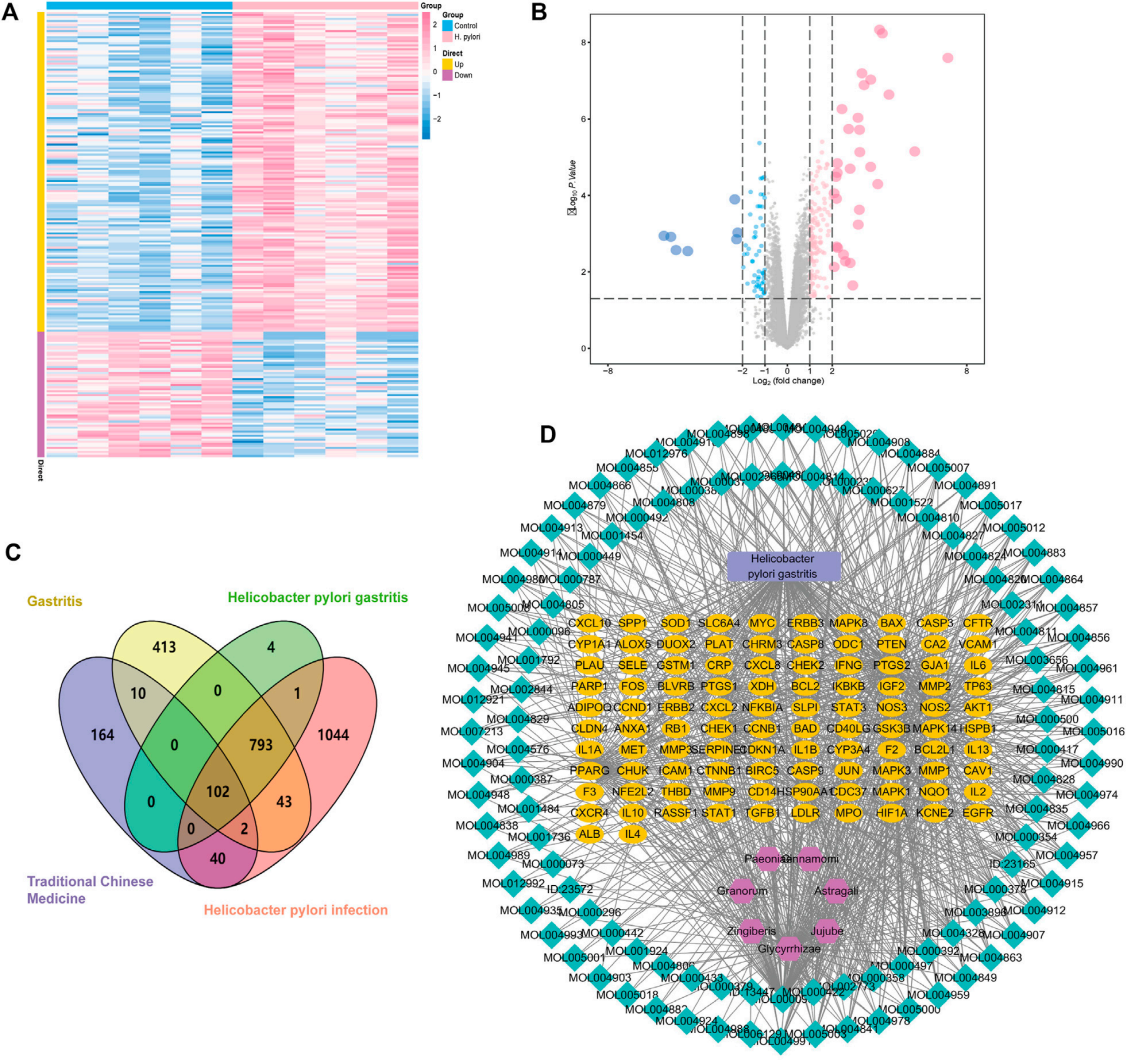
Potential targets of HQJZD in the treatment of *H. pylori* gastritis. **(A)** Cluster heat maps of the differential genes. **(B)** Volcano maps of the differential genes: pink represents the 647 significantly up-regulated genes and blue represents the 253 significantly down-regulated genes. **(C)** Venn diagram of disease targets from different databases and targets of HQJZD: purple represents the 318 targets of active components in HQJZD; yellow represents the 1363 targets of “gastritis”; and pink represents the 2025 targets of “*Helicobacter pylori* infection” from the GeneCards database. Green represents the 2025 targets of 900 differential genes from GEO database. Finally, 102 intersection targets of HQJZD in treating *H*. *pylori* gastritis were obtained. **(D)** The “active ingredients–disease–targets” network was constructed and included 224 nodes and 998 edges.

The series matrix file data file of GSE27411 was downloaded from the NCBI GEO public database using GPL6255 as the annotation platform, containing transcriptome data from 12 patients with gastritis, including six normal persons and six patients with *H. pylori* gastritis. With screening conditions of | logFC >1 | and *p* < 0.05, 900 differential genes (DEGs) were obtained between the two groups using a limma package analysis in R language. The DEGs contained 647 up-regulated and 253 down-regulated genes, and were used to draw cluster heat maps and volcano maps using R language. Here, pink represents the significantly up-regulated genes and blue represents significantly down-regulated genes (Figures 4A,B).

We used the terms, “gastritis” and “*Helicobacter pylori* infection” as keywords, and when retrieval was performed in the GeneCards database (https://www.genecards.org/), 1363 and 2025 relevant targets were obtained, respectively. Our search results were combined with the targets obtained from the GEO gene chip, and after taking the intersection, 895 disease-related gene targets were obtained. The selected drug and disease targets were added to the Venn diagram drawing software (Venny 2.1) to obtain 102 intersection targets for subsequent analysis as potential targets of HQJZD in treating *H. pylori* gastritis (Figure 4C).

Seven herbs, 128 active ingredients, and 102 intersection targets of HQJZD were imported onto the Cytoscape 3.7.2 software to establish the “active component-disease-target” network that included 224 nodes and 998 edges. The “nodes” indicate active ingredients and targets, and the “edges” indicate the connection between active ingredients and targets. Larger nodes act as regulatory hubs in the network and might be key compounds or targets (Figure 4D). Green nodes represent active ingredients, yellow nodes represent targets of drug actions on the disease, purple nodes represent traditional Chinese medicine, and blue rectangular nodes represent the disease.

According to the topological parameters calculated by the Network Analyzer, the six key active components of HQJZD, including quercetin, kaempferol, *β*-carotene, *β*-sitosterol, formononetin, and licochalcone A were screened out as the condition of degree value greater than 2 times. *β*-Carotene and licochalcone A were the only active components from *Fructus Jujube* and *Radix Glycyrrhizae* respectively, while the other active components were commonly found (Table 2) in two or more herbs of HQJZD. Among them, the degree of quercetin was 77, indicating its association with multiple targets. Furthermore, ALB, IL-6, AKT1, IL-1B, JUN, and other target proteins with high centrality were connected with multiple active components. Different active components may have synergistic effects and may reflect the characteristic multiple components and multiple targets of traditional Chinese medicine.

**TABLE 2:** Key active ingredients of HQJZD.

No.	Component	Structure	Betweenness centrality	Closeness centrality	Degree
1	Quercetin	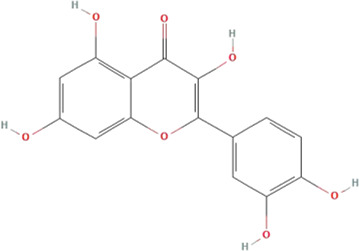	0.184357	0.55335	77
2	Kaempferol	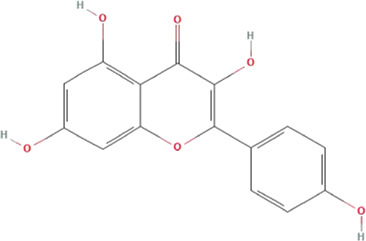	0.035062	0.441584	27
3	β-Carotene	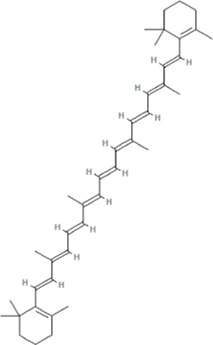	0.008881	0.416822	17
4	β-Sitosterol	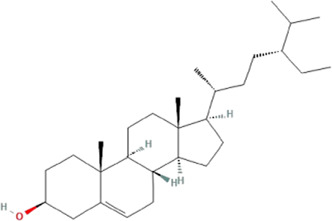	0.017688	0.42155	16
5	Formononetin	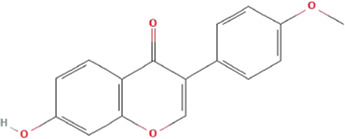	0.005887	0.419962	15
6	Licochalconea	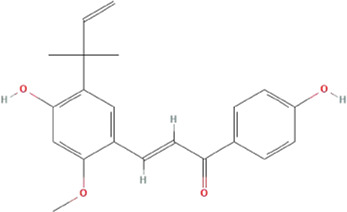	0.009589	0.419962	15

#### Construction of PPI network and enrichment analysis

**FIGURE 5:**
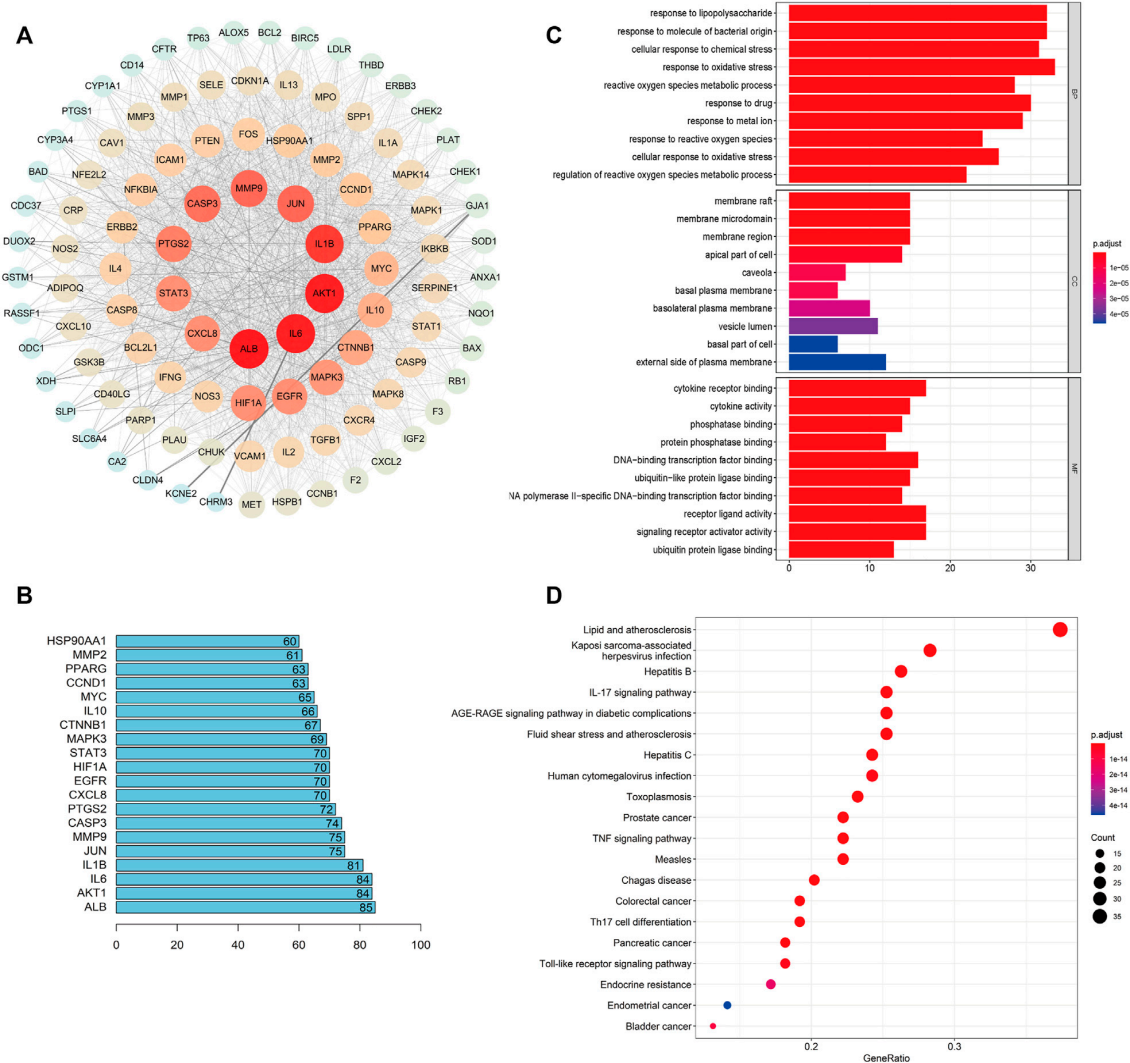
PPI Network and GO and KEGG enrichment analysis. **(A)** PPI network of the 102 intersection targets constructed using Cytoscape. The size of nodes represents their degree values. **(B)** The top 20 key targets of HQJZD in treating *H. pylori* gastritis. **(C)** All top 15 significantly enriched terms in molecular function (MF), biological process (BP), and cellular component (CC). **(D)** Top 20 significantly enriched terms in the KEGG pathway.

The PPI network of HQJZD is shown in Figure 5A and involves 102 nodes with 1975 edges having an average degree of 38.7. The color and size of the nodes are adjusted according to the degree value. The more red the diameters and the more intense the colors of the nodes, the higher the degree they represent. Coarser connection lines are synonymous with stronger interactions. A network topology analysis according to the degree ranking indicates that genes with a higher score than average are selected as key targets, and a total of 50 key targets are screened, and the top 20 targets visualized, with the abscissa as the degree of each target (Figure 5B; Table 3).

**TABLE 3:** Top 20 key targets of Huangqi Jianzhong decoction.

No.	Uniprot ID	Target gene	Target protein	Betweenness centrality	Closeness centrality	Degree
1	P02768	ALB	Albumin	0.04760741	0.86956522	85
2	P05231	IL-6	Interleukin-6	0.05872245	0.86206897	84
3	P31749	AKT1	RAC-alpha serine/threonine-protein kinase	0.0444836	0.86206897	84
4	P01584	IL-1B	Interleukin-1 beta	0.03482047	0.84033613	81
5	P05412	JUN	Transcription factor Jun	0.0205545	0.8	75
6	P14780	MMP9	Matrix metalloproteinase-9	0.02253655	0.8	75
7	P42574	CASP3	Caspase-3	0.01748282	0.79365079	74
8	P35354	PTGS2	Prostaglandin G/H synthase 2	0.01821789	0.78125	72
9	P40763	STAT3	Signal transducer and activator of transcription 3	0.01101789	0.76335878	70
10	P10145	CXCL8	Interleukin-8	0.01622582	0.76335878	70
11	Q16665	HIF1A	Hypoxia-inducible factor 1-alpha	0.01469114	0.76923077	70
12	P00533	EGFR	Epidermal growth factor receptor	0.02840032	0.76923077	70
13	P27361	MAPK3	Mitogen-activated protein kinase 3	0.01645888	0.76335878	69
14	P35222	CTNNB1	Catenin beta-1	0.02263825	0.7518797	67
15	P22301	IL-10	Interleukin-10	0.01213143	0.74074074	66
16	P01106	MYC	Myc proto-oncogene protein	0.01639746	0.73529412	65
17	P37231	PPARG	Peroxisome proliferator-activated receptor gamma, PPAR-gamma	0.01275376	0.72463768	63
18	P24385	CCND1	G1/S-specific cyclin-D1	0.01419865	0.72992701	63
19	P08253	MMP2	Matrix metalloproteinase-2	0.00763925	0.71942446	61
20	P07900	HSP90AA1	Heat shock protein HSP 90-alpha	0.01471284	0.71428571	60

The common targets of drug diseases were enriched for the biological process (BP), molecular function (MF), and cellular component (CC) of GO enrichment analysis, resulting in a total of 2254 BP, 106 related MF, and 53 related CC. According to the size of the -log *p* value, the top 10 items sorted by each module were selected for visualization using R language (Figure 5C). The biological processes mainly involved metabolic regulation; the reactive oxygen species metabolic process; response to drugs; and response to lipopolysaccharides, molecules of bacterial origin, drugs, metal ions, oxidative stress, chemical stimulation, etc. Cellular components mainly included cell membrane microdomains, membrane rafts, membrane domains, cell tips, cell basal membrane, basal cells, and lateral cytoplasmic membrane. Molecular functions mainly included cytokine receptor binding, enzyme binding, transcription factor binding, cytokine activity, and signal receptor agonist activity.

We obtained 159 signaling pathways using the KEGG signaling pathway enrichment analysis, and the top most significant 20 pathways were selected to draw the pathway enrichment analysis map (Figure 5D). Results show that HQJZD was mainly involved in signaling pathways of atherosclerosis, AGE-RAGE, T-cell receptors, TNF-α, IL-17, toll-like receptors, hepatitis C, pancreatic cancer, colorectal cancer, bladder cancer, toxoplasmosis, and others, in treating *H. pylori* gastritis.

#### Molecular docking validation

**FIGURE 6:**
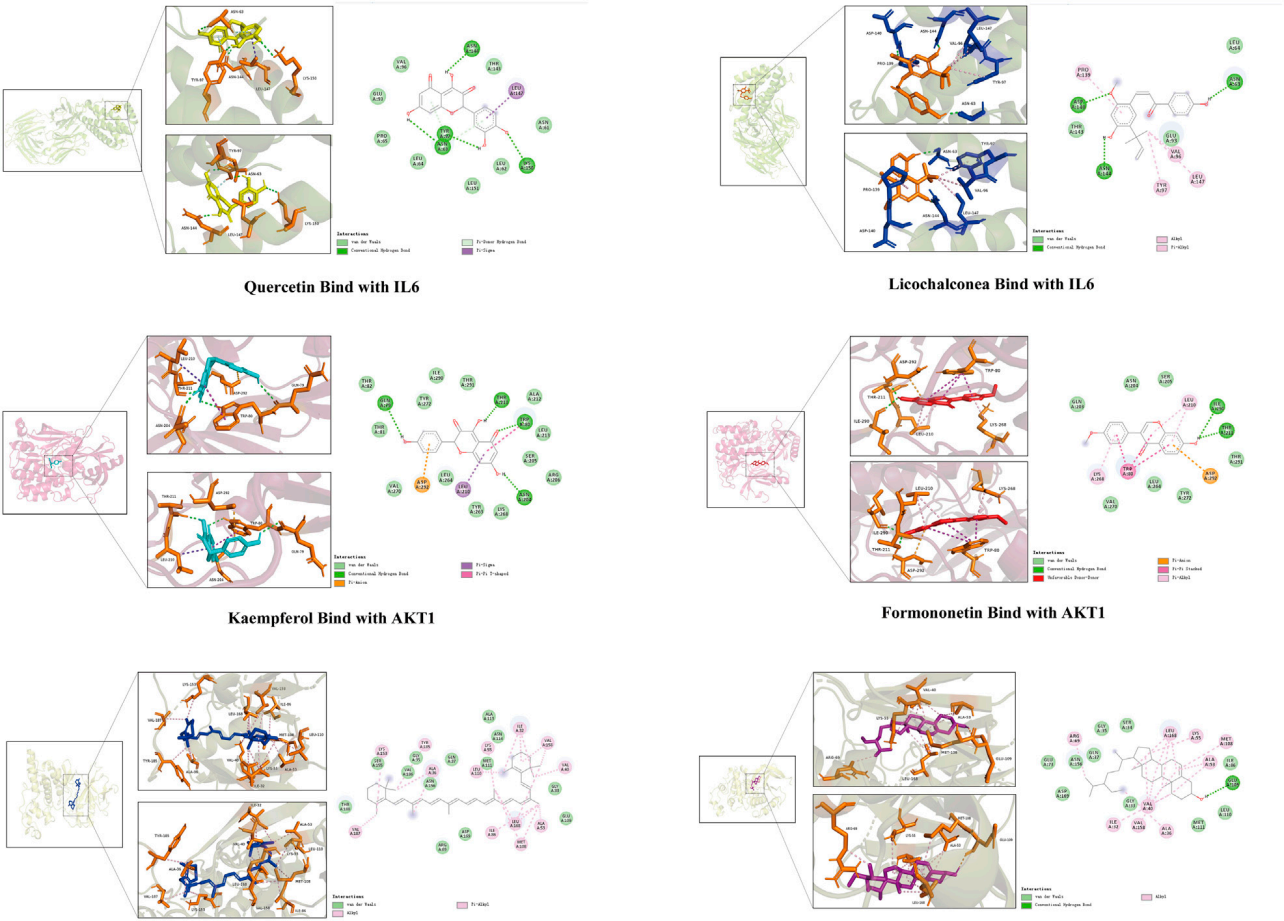
Interaction between key components of HQJZD and core target proteins. The six figures exhibit the quercetin and licochalconea binding with IL-6; kaempferol and formononetin binding with AKT1; and *β*-carotene and *β*-sitosterol binding with JUN.

The six key active ingredients selected from the “active ingredient–disease–target” network diagram (mistletoe bombesin, carabinol, *β*-carotene, *β*-sitosterol, formononetin, and licochalcone A) were docked one by one with five key proteins: ALB (PDB ID: 4LB2), IL-6 (PDB ID: 4O9H), AKT1 (PDB ID: 6S9W), IL-1B (PDB ID: 1T4Q), and JUN (PDB ID: 4QTD) in the PPI network diagram using the Autodock Vina software. The binding energy of <0 kcalmol^−1^ is considered when molecular protein spontaneously binds and interacts, and lower binding energies are related to higher stability in molecular conformations. A binding force of < −5.0 kcal·mol^−1^ indicates the binding force between the two is strong. Docking results were imported onto the Pymol software for visualization, and the binding of the six key components to key targets is shown in Table 4 and Figure 6. The target protein is represented by different colors, the key residues (hydrophobic interaction residues and residues forming hydrogen bonds) are represented by lines, and the ligands are represented by different colored sticks. Results show that binding energies of 5 core proteins and 6 key components were ≤ −5 kcal/mol, which indicates the key active components of HQJZD that can bind well to target proteins.

**TABLE 4:** Docking binding free energy of key components to core target molecules (kcalmol^−1^).

No.	Compound	Binding energies				
		ALB	IL-6	AKT1	IL-1B	JUN
1	Quercetin	−7.55	−11.33	−10.88	−5.71	−10.68
2	Kaempferol	−6.27	−9.36	−11.2	−5.96	−11.1
3	β-Carotene	−6.34	−7.03	−7.65	−5.34	−8.31
4	β-Sitosterol	−5.16	−6.53	−7.6	−5.7	−7.76
5	Formononetin	−6.04	−7.32	−8.05	−6	−7.85
6	Licochalconea	−5.91	−7.5	−7.47	−5.83	−7.31

## Discussion

Gastric cancer is becoming increasingly common globally. Less than 20% of patients with gastric cancer survive for >5 years after diagnosis (Tsai et al., 2022). *H. pylori*, a Gram-negative bacterium, plays an important role in the pathogenesis of gastric cancer (Navashenaq et al., 2021). About 60% of gastric cancer patients from developed countries, and 75% of patients with gastric cancer from developing countries suffer from chronic *H. pylori* infection (Park et al., 2021). Chronic gastritis caused by *H. pylori* infection results in the release of pathogenic factors such as urease, vacuolar toxin A, CagA protein, inflammatory mediators, and reactive oxygen species metabolites, which lead to abnormal proliferation and apoptosis of gastric mucosal epithelial cells, resulting in gastric cancer (Noda et al., 2021). Although recent progress with antibiotics and other drugs has been made in treating *H. pylori* infection, it remains an important factor in increasing the incidence of gastric cancer (Ding, 2020). Therefore, to improve the cure rate after *H. pylori* infection, it is particularly important for clinical prevention and treatment that we understand the molecular mechanisms for the proliferation and apoptosis of human GES-1 cells after such infection. Our study established a human GES-1 cell model infected with *H. pylori*, and detection of relevant indicators indicated that this infection reduces the survival rates of human GES-1 cells and increases the level of apoptosis, hence leading to stomach-related diseases. Intervention with HQJZD significantly improves cell viability, inhibits cell apoptosis, and reduces the expression of *H. pylori-*related virulence factors, such as HpPrtC, OPiA, IceA1, and BabA2.

Traditional Chinese medicine hypothesizes that *H. pylori*-associated gastritis belongs to the categories of “epigastric pain,” “acid swallowing,” “vomiting,” “noise,” “hiccups,” and “fullness” (Lu et al., 2021). In recent years, an in-depth study found that most *H. pylori* infection belongs to the spleen and stomach deficiency cold type, spleen and stomach weakness leads to blockage in the reception and transport of water, causing stomach pain, bloating, fullness, and other symptoms. *H. pylori* belongs to the “damp-heat pathogen” in traditional Chinese medicine, and acts as a poison, causing weakness of spleen and stomach, a damp-heat barrier, qi stagnation and blood stasis, and stagnation of liver-qi, as it invades the stomach and leads to gastritis (Wang, 2019). Our product is used clinically to treat chronic gastritis, peptic ulcer, atrophic gastritis, and other gastrointestinal diseases, with a significant effect on *H. pylori* gastritis. *Radix Astragali* is a monarch drug in a formula that is able to enhance the phagocytic function of the human immune system, scavenge free radicals, inhibit the growth of *H. pylori*, promote gastric mucosal repair, and produce anti-tumor effects (Wu et al., 2022). *Paeoniae Radix Alba*, *Ramulus Cinnamomi*, and *Radix Glycyrrhizae* are minister drugs, of which *Glycyrrhiza uralensis* can reduce gastric acid concentration and inhibit bacteria (Luo et al., 2022); and *Ramulus Cinnamomi*, combined with *Paeoniae Radix Alba* and *Fructus Jujube*, can invigorate qi, warm the center of the body and dissipate cold. It can tranquilize the mind and nourish the blood, and even improve edema and relieve pain (Nöst et al., 2019). The entire prescription has the effect of clearing away heat and nourishing yin; activating blood circulation and supplementing qi; and significantly improving the body’s immunity. Animal experiments have confirmed that Huangqi Jianzhong decoction can regulate serum gastrin levels and inhibit the secretion of gastric acid and pepsinogen in rats with spleen deficiency (Sun et al., 2005). Clinical studies have shown that HQJZD significantly improves gastroscopy results and the *H. pylori* clearance rate, increases levels of substance *P* in the antrum, and promotes gastric emptying in patients (Wei et al., 2015). In addition, some studies have shown that Huangqi Jianzhong decoction can promote the growth of probiotics, such as bifidobacteria and lactic acid bacteria, inhibit harmful bacteria, and enhance the therapeutic effect on gastrointestinal diseases by regulating the intestinal flora environment, so as to exert its efficacy (Jin, 2021). However, the specific molecular mechanism of HQJZD in treating *H. pylori* gastritis is still unclear and needs further study.

We observed the key active components of 7 herbs in HQJZD, which included quercetin, kaempferol, *β*-carotene, *β*-sitosterol, formononetin, and licochalconea, all flavonoids, based on network pharmacology screening. Studies have shown that flavonoids can produce an antibacterial effect on *H. pylori*-induced human gastric cancer cells (AGS) by releasing inflammatory factors such as IL-8 (Skiba et al., 2016). Quercetin is a common component of *Radix Astragali*, *Radix Glycyrrhizae*, and *Fructus Jujube* in HQJZD and has the highest moderate value in the “component–disease–target” network. It can prevent gastritis by affecting p38 MAPK, Bcl-2, and BAX levels, and can regulate the balance of gastric cell proliferation and apoptosis (Zhang et al., 2017a). Kaempferol, present in *Radix Paeoniae Alba*, *Radix Astragali*, and *Radix Glycyrrhizae*, can significantly inhibit the cytotoxin-associated gene A (CagA) and vacuolating cytotoxin A (VacA) of *H. pylori*, and can reduce expression of proinflammatory cytokines and inhibit infection (Yeon et al., 2019). Formonetin and licochalcone A both have protective effects on the gastric mucosa (Mendonça et al., 2020).

We screened 50 key targets by protein-protein interaction, and based on degree value ranking: more important targets included ALB, IL-6, AKT1, IL-1B, and JUN. Recent studies show that IL-6, IL-1β, and TNF-α are three typical proinflammatory cytokines (Tourani et al., 2018). These proinflammatory cytokines are up-regulated when infected with *H. pylori*. Among them, IL-6 activates the STAT3 pathway, and IL-1β can reduce gastric acid secretion, ultimately leading to the development of *H. pylori* gastritis or even gastric cancer (Zhang et al., 2017b). AKT1 is one of three closely related serine/threonine protein kinases (AKT1, AKT2, and AKT3) called AKT kinases, which regulate many processes, including metabolism, proliferation, and cell survival, growth, and angiogenesis. Studies have shown that *H. pylori* promotes gastric epithelial cell survival and participates in early tumorigenesis through the PLK1/PI3K/Akt pathway (Xu et al., 2018). Results of pathway enrichment analysis show lipid and atherosclerotic pathways, IL-17 signaling pathways, AGE-RAGE receptor signaling pathways, TNF signaling pathways, and some cancer signaling pathways. *H. pylori* pathogenicity results from a combination of host, environmental, and bacterial virulence factors, and maintains the bacterial integrity dependent on the host’s cholesterol content (Shimomura et al., 2013). Other studies confirm that cholesterol-rich atherosclerotic plaques promote the survival and growth of *H. pylori* (Testerman et al., 2019). HQJZD may inhibit *H. pylori* activity by regulating lipids and atherosclerotic pathways. Both TNF-α and NF-κB play key roles in inflammation and cancer development, and the *H. pylori*-specific virulence factor, Tip-α, is a TNF-α-inducing protein that participates in TNF-α and NF-κB pathways as an oncogenic factor mediating inflammation and immune responses, and has an important part in triggering chronic gastritis, hyperplasia, and gastric cancer (Morningstar-Wright et al., 2022).

In summary, our work establishes a human GES-1 cell model infected by *H. pylori* and finds that HQJZD can effectively increase the proliferation rate of GES-1 cells and inhibit apoptosis, and that it has significant anti-*H. pylori* activity. Network pharmacology analysis reveals components of quercetin, kaempferol, *β*-carotene, *β*-sitosterol, formononetin, and licochalconea may be pharmacodynamic substances of HQJZD for treating *H. pylori* gastritis. The anti-*H. pylori* mechanism of HQJZD may involve multiple targets, pathways, and biological processes. These findings provide the scientific basis for the clinical application of Huangqi Jianzhong decoction against *H. pylori.*

## Data Availability

The original contributions presented in the study are included in the article/Supplementary Material. Further inquiries can be directed to the corresponding author.
